# The effect of oral care intervention on pneumonia hospitalization, *Staphylococcus aureus* distribution, and salivary bacterial concentration in Taiwan nursing home residents: a pilot study

**DOI:** 10.1186/s12879-020-05061-z

**Published:** 2020-05-27

**Authors:** Tien-Cheng Chiang, Ming-Shyan Huang, Po-Liang Lu, Shun-Te Huang, Ying-Chu Lin

**Affiliations:** 1 School of Dentistry, College of Dental Medicine, Kaohsiung Medical University, No.100, Shiquan 1st Rd., Sanmin Dist., Kaohsiung City, 807 Taiwan; 2 Division of Pulmonary and Critical Care Medicine, Department of Internal Medicine, E-DA Cancer Hospital, No. 21, Yida Rd., Yanchao Dist., Kaohsiung City, 824 Taiwan; 3 Division of Infectious Diseases, Department of Internal Medicine, Kaohsiung Medical University Hospital, No.100, Tzyou 1st Rd., Sanmin Dist., Kaohsiung City, 807 Taiwan; 4 Division of Special Care Dentistry, Department of Dentistry, Kaohsiung Medical University Hospital, No.100, Tzyou 1st Rd., Sanmin Dist., Kaohsiung City, 807 Taiwan

**Keywords:** Dental care, Nursing homes, Oral health, Pneumonia, Saliva, *Staphylococcus aureus*

## Abstract

**Background:**

Elevated *Staphylococcus aureus* and oral bacterial concentrations are known to correlate with pneumonia hospitalization in nursing home residents. However, the effects of a professional oral care intervention on these factors remain unclear. The aims of this quasi-experimental study were to compare bacterial concentrations in saliva and sputum, oral health status, distribution of *Staphylococcus aureus*, and pneumonia status before and after a professional oral care intervention.

**Methods:**

A purposive sample of residents from two nursing homes was divided into an intervention group that received a weekly professional oral care intervention and a control group. Oral bacterial concentration was determined by real-time polymerase chain reaction. The *Staphylococcus aureus* distribution was determined by bacterial culture and matrix-assisted laser desorption/ionization–time of flight mass spectrometry. After data collection, a statistical analysis was performed to evaluate the effect of the intervention.

**Results:**

Most residents were unconscious (80%), and most had a history of pneumonia (76%). Baseline demographic data did not significantly differ between the two groups. After the intervention, the intervention group had significant improvements in plaque index (1.66 ± 0.78 vs. 0.94 ± 0.64, *p* <  0.01), gingival index (2.36 ± 0.76 vs. 1.65 ± 0.83, *p* <  0.01), tongue coating index (0.96 ± 1.10 vs. 0.16 ± 0.47, *p* <  0.01), distribution of *Staphylococcus aureus* in salivary samples (11.11 ± 14.47% vs. 1.74 ± 3.75%, *p* = 0.02), and salivary bacterial concentration ([4.27 ± 3.65] × 10^5^ vs. [0.75 ± 1.20] × 10^5^, *p* <  0.01). Sputum bacterial concentration did not significantly differ. The intervention group also had a significantly lower annual prevalence of pneumonia hospitalization (1.24 ± 1.51 vs. 0.48 ± 0.59, *p* = 0.01), especially in residents whose salivary bacterial concentration exceeded the median. However, the duration of pneumonia hospitalization did not significantly differ between the two groups.

**Conclusion:**

A professional oral care intervention in nursing home residents can improve oral health, reduce levels of salivary bacteria and *Staphylococcus aureus*, and decrease the annual prevalence of pneumonia hospitalization.

**Trial registration:**

Trial registration: ClinicalTrials.gov, NCT03874962. Registered 12 March 2019 - Retrospectively registered.

## Background

Population aging is a global phenomenon [1]. In 2018, the elderly population of Taiwan comprised 14.56% of the total population, which met the criterion for an “aged society” [2]. Pneumonia is a major health problem that can be fatal in elderly people, especially those who are nursing home residents (NHRs) [3]. In 2016, pneumonia was the third leading cause of death in Taiwan [4]. Notably, 91% of all pneumonia deaths that year occurred in individuals older than 65 years [4]. In the elderly population, the pneumonia hospitalization risk is 1.96 to 10 times higher in NHRs compared to those who live in the community [5, 6]. Poor oral health has an important role in the local infection disease and pneumonia development in NHRs [7]. Therefore, elderly NHRs with poor oral health status are at high risk of developing pneumonia.

Pneumonia is a critical and potentially fatal disease in NHRs [3, 8]. Inadequate oral care has been identified as a potential risk factor for pneumonia, especially in NHRs [9]. A Japan study reported that NHRs with poor oral hygiene had higher salivary bacterial concentrations compared to those with good oral hygiene [10]. Studies show that pneumonia is often associated with specific bacteria and bacterial loads in the oral cavity [3, 8, 11]. A common cause of pneumonia is colonization of the oropharynx by *Staphylococcus aureus* (*S. aureus*), aerobic Gram-negative bacilli, and *Pseudomonas aeruginosa* [12]*.* A previous study of pneumonia in residents of long-term care facilities reported that the major isolates were aerobic pathogens of *S. aureus* (45%) followed by enteric Gram-negative bacilli (42%) and *Pseudomonas aeruginosa* (13%) [13]. The authors reported that dental plaque colonization preceded oropharyngeal colonization and acted as a potential reservoir for lower respiratory tract infection and the development of pneumonia [13]. Researchers in the United States reported a 28.6% prevalence of *S. aureus* on environmental surfaces in seven nursing homes [14]. *S. aureus* is primarily a nosocomial infection acquired in institutions such as nursing homes. *S. aureus* can spread directly between individuals or indirectly through fomites.

Oral care interventions in previous studies have focused on care of the teeth, gums, palate, buccal mucosa, and tongue [3, 8, 15, 16]. For example, one study reported that integrating salivary gland massage in an oral health program for elderly people can increase the flow rate of salivary glands [17]. However, few studies have investigated how an oral care intervention that includes massage of the salivary glands affects pneumonia, oral bacterial levels, and *S. aureus* infection in NHRs.

Therefore, this study designed and implemented a professional oral care intervention (POCI) in a population of elderly NHRs and then compared distributions of *S. aureus*, bacterial concentrations in saliva and sputum, and pneumonia hospitalization before and after the intervention.

## Methods

### Study design and setting

**Fig. 1:**
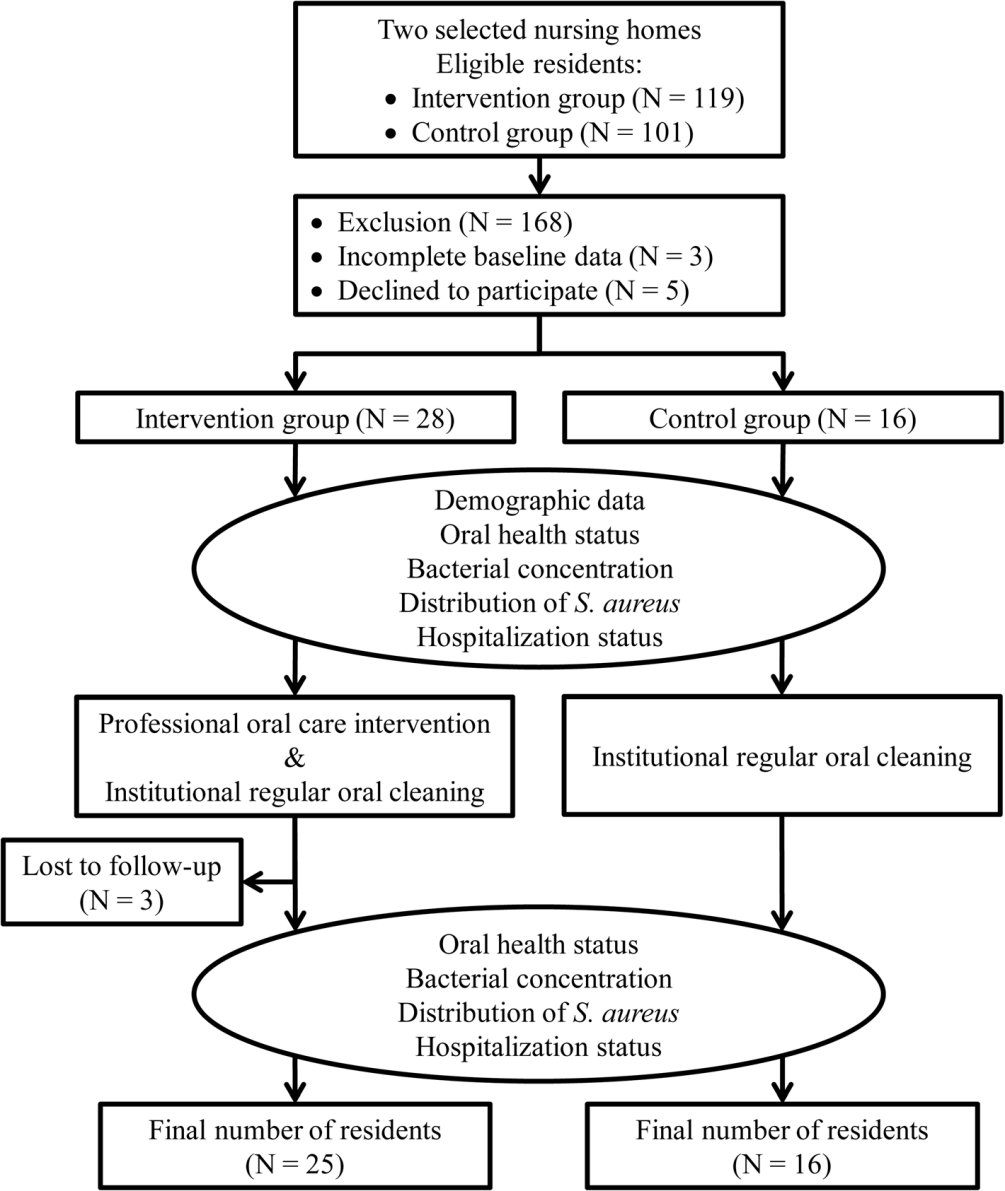
Flowchart of study procedure

Figure 1 is a flowchart of the procedure for this quasi-experimental study, which was performed from August, 2015 to December, 2017. In August, 2015, a purposive sample of residents from two nursing homes in Kaohsiung, Taiwan, was divided into an intervention group and a control group. Both nursing homes were affiliated with regional hospitals, and both had a capacity exceeding 80 residents. From June to September of 2016, the baseline data including the standardized questionnaire survey, medical records of pneumonia history, oral health examination, and salivary and sputum samplings were collected at 9:00–11:00 am before the first intervention. The POCI was performed once a week (8:00–10:00 am) from October to December of 2016. The POCI required 30 min to complete in each resident. Within 1 h after the last POCI, each participant in the intervention group was collected the data including the standardized follow-up questionnaire, medical records of pneumonia history, oral health examination, and salivary and sputum samplings. The control group were also assessed these data during 9:00–11:00 am after their regular oral cleaning.

### Participants

The eligible population of intervention and control groups were 119 and 101, respectively. The inclusion criteria were as follows: bedridden for ≥6 months, aged over 50 years, dysphagia, and able to produce sputum. The number of participants recruited in intervention and control groups were 30 (25.2%) and 22 (21.8%), respectively. Out of 52 residents who met the inclusion criteria, three were excluded because baseline data were incomplete, and five residents or their healthcare surrogates declined to participate. The final number of residents in the intervention group and the control group were 28 and 16, respectively (Fig. 1). The study protocol was approved by the Institutional Review Board of Kaohsiung Medical University Hospital (KMUH), Taiwan (ID number: KMUHIRB-SV-20150003(I)) and ClinicalTrials.gov (ID number: NCT03874962) [18].

### Questionnaire and oral examination

After obtaining written informed consent from the participants, the researchers collected data from medical records, including sociodemographic data, status of consciousness, medical diagnosis of dysphagia and pneumonia, and the annual prevalence and duration of pneumonia hospitalization. Data for pneumonia history were collected from 1 year before the intervention until the follow up at 1 year after the intervention. Residents with a Glasgow Coma Scale [19] score under 8 points were classified as unconscious. While blinded to all NHR data, two well-trained dentists in the Division of Special Care Dentistry (kappa = 0.83) performed all clinical assessments of dysphagia and oral health examinations and collected all data for plaque index (PI) [20], gingival index (GI) [21], and tongue coating index (TCI) [22]. The PI indicates plaque accumulation visible to the naked eye and ranges from 0 (no plaque) to 3 (abundant plaque in teeth and gingival margin). The GI indicates gingival inflammation and ranges from 0 (normal) to 3 (severe). The TCI indicates tongue coating (bacteria, saliva, food debris, exfoliated epithelial cells, and exuded leukocytes) coverage and ranges from 0 (no coating) to 4 (thick coating covering more than two thirds of the tongue).

### Laboratory methods

#### Salivary and sputum samples

A salivary sample was collected using a sterilized transwab (Creative Biotechnology Co., Ltd., Taiwan). The bacteria sample was then released into an Eppendorf tube containing 1.5 mL of normal saline. A mucus extractor (Pacific Hospital Supply Co. Ltd., Taiwan) was used to draw a 1-mL sample of sputum. The viscosity of the sputum sample was decreased by adding an equal volume of 30.67 mM of N-acetyl-L-cysteine (BBL MycoPrep) solution to the sample at room temperature for 60 min.

#### Bacterial concentration measurement of standard curve, salivary sample, and sputum sample

**Fig. 2:**
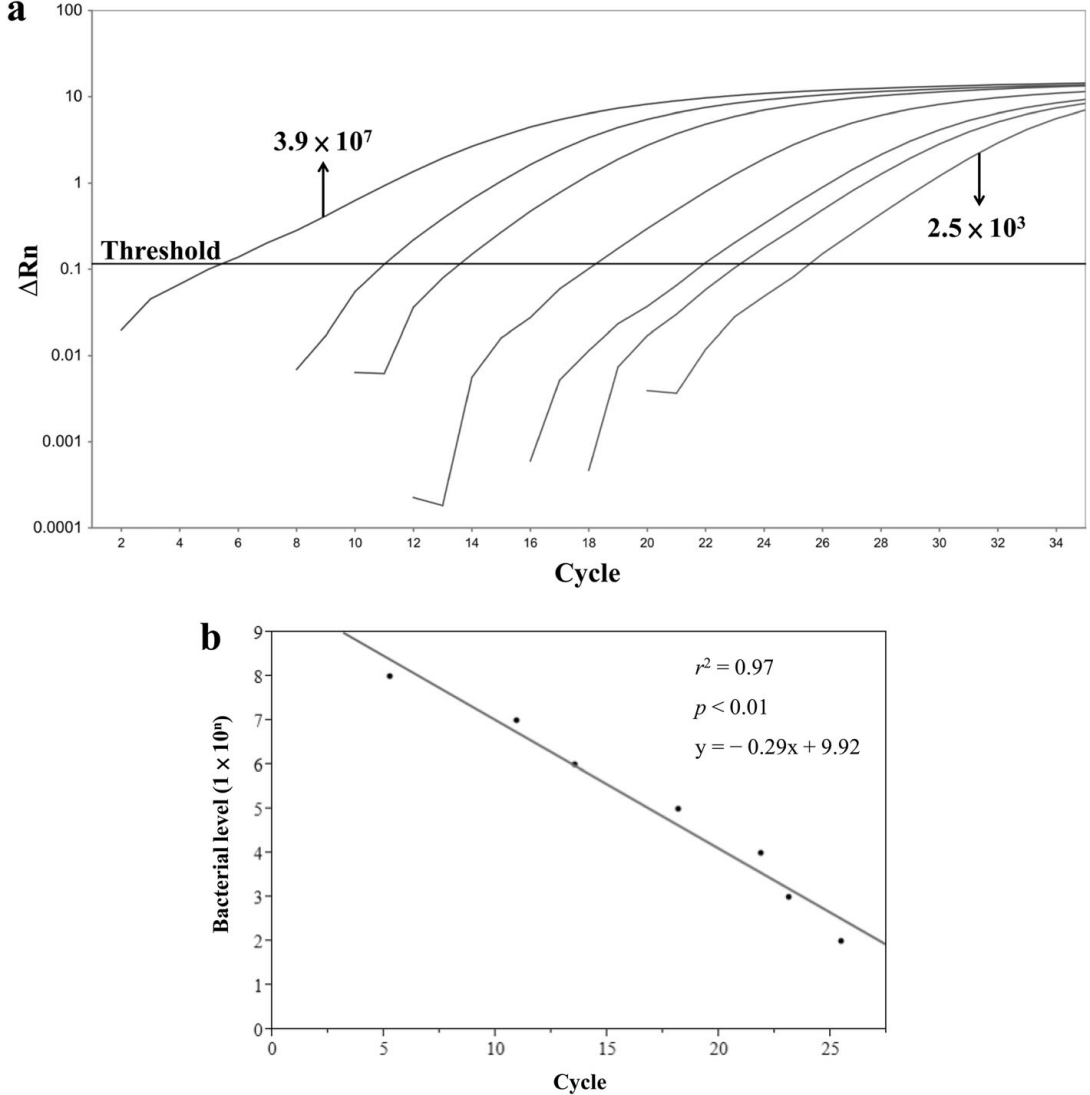
Results of real-time polymerase chain reaction analysis of fivefold serial dilution of standard bacterial DNA. (**a**) Amplification plots. (**b**) Linear regression reference

The pure *S. aureus* strain (strain ID: ATCC 29213) was used as the positive control in bacterial identification. After overnight culture, the *S. aureus* samples were prepared by fivefold serial dilution and plated in a Petri dish with suitable agar medium to produce from 2.5 × 10^3^ to 3.9 × 10^7^ colony-forming units/mL. A modification of the standard method [23] was used to extract genomic DNA from 1 mL of each bacterial serial dilution of each salivary and sputum sample. After DNA concentrations were determined, the samples were stored at − 80 °C until real-time polymerase chain reaction (RT-PCR) was performed. The RT-PCR target was 16S rRNA gene. Identified *S. aureus* was verified by 16S rRNA PCR direct sequencing. The standard curve for bacterial concentration (Fig. 2) was generated using the genomic DNA from a fivefold serial dilution and their RT-PCR threshold cycle by the StepOnePlus System (Applied Biosystems). Protocols for the RT-PCR assay were performed as described previously [24]. The universal forward primer sequence [25] was 5′-CCT ACG GGA GGC AGC AG-3′, and the reverse primer sequence [26] was 5′-CCG TCA ATT CMT TTR AGT TT-3′. The coverages of common bacteria for forward primer and reverse primer were 94.9 and 92.8%, respectively [27]. Previous study also reported the primer pair with no obvious bias toward the majority of bacterial species [27]. After the regression equation was created from the standard curve, bacterial concentration of each salivary and sputum sample was estimated using this reference line equation and their RT-PCR results. The coefficient of variation in the threshold cycle value for three duplications of RT-PCR was between 2 and 12%. Human genomic DNA from blood was used as the negative control.

#### Identification of bacterial species

To identify species of oral bacteria, 10 μL of salivary samples and sputum samples were cultured in Bacto Agar (Becton, Dickinson and Company, New Jersey, US) culture media. After incubation at 37 °C for 16 h, 24 single colonies were randomly selected to identify bacterial species in the salivary and sputum samples. After a pure culture of each colony, three single colonies were reselected and transferred to the mesh of a matrix-assisted laser desorption/ionization–time of flight mass spectrometer (MALDI-TOF MS) (Bruker Corporation, Massachusetts, US) to identify the bacterial species. No discrepancy in bacterial identification was found among these three single colonies. The MALDI-TOF MS device had 96 and 93% sensitivity in identifying the genus and species, respectively, of common bacterial isolates [28]. In total, 98 oral bacterial species were identified from the originally and randomly selected single colonies. However, only six identified bacterial species were recognized as pneumonia pathogens [15, 29, 30] in all bacterial samples before POCI: *S. aureus* (12.7%), *Pseudomonas aeruginosa* (8.9%), *Acinetobacter baumannii* (4.1%), *Streptococcus pneumoniae* (0.8%), *Haemophilus influenzae* (0.7%), and *Klebsiella pneumoniae* (0.3%). After considering the sample size of participants, the number of randomly and originally selected single colonies (*n* = 24), the statistical power (> 0.6), and the difference in frequency distributions of oral bacterial species between before and after intervention, *S. aureus,* the only pneumonia pathogen with a distribution larger than 5% was selected for further analysis.

### POCI

Institutional oral cleaning was regularly performed by caregivers one or two times daily with a toothbrush or foam swabs. In addition to regular oral cleaning, POCI was performed in the intervention group by dental hygienists once a week for 3 months. Ten dental hygienists performed the POCI in the intervention group. All dental hygienists had been certified by the Division of Special Care Dentistry, KMUH, Taiwan, after completing training and passing an examination. For residents, the POCI included facial muscle massage, salivary gland massage, toothbrush care, and soft tissue cleaning. For family members and caregivers, the POCI included oral health education and consultation. The purpose of the facial muscle massage was to relax muscles in the neck, cheeks, and lips and to reduce tension. The salivary gland massage was performed to improve the salivary section in the parotid gland, submandibular gland, and sublingual gland. Toothbrushes, end-tufted brushes, interdental brushes, and dental floss were used to remove food debris and dental plaque on tooth surfaces, especially on crowded teeth, on tooth surfaces next to missing teeth, and around bridges and other dental prostheses. Soft tissue cleaning was performed with foam swabs to clean the palate, tongue, sublingual mucosa, and buccal mucosa. To avoid choking, a suction tool was used to remove contaminated liquid from the oral cavity. Use of oral moisturizers at least four times daily was prescribed for residents with dry mouth. Alcohol-free mouthwash solution (China Chemical & Pharmaceutical Co., Ltd., Taiwan) containing chlorhexidine and sodium monofluorophosphate was used for toothbrush care and soft tissue cleaning. The intervention was performed without toothpaste so that toothpaste foam would not interfere with oral cleansing. After 1 year of POCI in the intervention group, the same POCI protocol was implemented in the control group.

### Statistical analysis

Since the sample size was not large, nonparametric tests for all statistical analysis were performed with JMP statistical software (version 9, SAS Institute Inc. Cary, North Carolina, U.S.). Fisher’s exact test was used to compare the frequency distributions of categorical data between the intervention and control groups. Wilcoxon rank-sum test was used to compare between-group differences in PI, GI, TCI, total bacterial concentration, *S. aureus* distribution, and pneumonia hospitalization status. Wilcoxon signed-rank test was used to compare differences in these variables before and after POCI. Kruskal-Wallis test and post hoc Tukey test were used to compare differences in bacterial concentration among three or more different groups. A *p*-value < 0.05 was considered statistically significant.

## Results

Three residents in the intervention group were lost to follow-up at the end of this study. Therefore, the final analysis included 25 and 16 residents in the intervention and control groups, respectively. Most residents were unconscious, and most had a history of pneumonia. Table 1 indicates that the groups did not significantly differ in demographic and clinical characteristics, including gender, age, history of pneumonia, and history of chronic disease. According to the results of Fisher’s exact test, the baseline frequency distribution of pneumonia hospitalization history in intervention group (*p* = 0.21) and control group (*p* = 0.49) who had received regular oral cleaning once a day did not significantly differ from those who had received regular oral cleaning twice a day, respectively. Additionally, baseline salivary bacterial concentration did not significantly differ among participants with TCI = 0 ([3.29 ± 3.22] × 10^5^ (*N* = 25)), participants with TCI = 1 ([3.80 ± 2.73] × 10^5^ (*N* = 5)), and participants with TCI ≥ 2 ([4.37 ± 4.72] × 10^5^ (*N* = 11)) (*p* = 0.72 in Kruskal-Wallis test). When PI and TCI were combined, baseline salivary bacterial concentrations significantly differed among group H (PI > 2 and TCI > 2: [5.18 ± 4.67] × 10^5^), group M (2 ≥ PI > 0 and/or 2 ≥ TCI > 0: [3.31 ± 2.39] × 10^5^), and group L (PI = 0 and TCI = 0: [1.28 ± 0.93] × 10^5^) in Kruskal-Wallis test (group H > group L, *p* = 0.03 in post hoc Tukey test).

**Table 1:** Demographic data for intervention and control groups

Characteristics	Intervention	Control	
	***N*** = 25 (%)	***N*** = 16 (%)	***p***-value^c^
Gender			0.34
Male	14 (56.0)	6 (37.5)	
Female	11 (44.0)	10 (62.5)	
Age^a^			0.53
≥ 74 years old	11 (44.0)	9 (56.3)	
< 74 years old	14 (56.0)	7 (43.7)	
Glasgow Coma Scale score			0.12
≥ 8 points	7 (28.0)	1 (6.3)	
< 8 points	18 (72.0)	15 (93.7)	
Education level			0.34
≥ high school	14 (56.0)	6 (37.5)	
< high school	11 (44.0)	10 (62.5)	
History of pneumonia			1.00
Yes	19 (76.0)	12 (75.0)	
No	6 (24.0)	4 (25.0)	
Diabetes			0.33
Yes	9 (36.0)	9 (56.3)	
No	16 (64.0)	7 (43.7)	
Hypertension			0.31
Yes	16 (64.0)	13 (81.3)	
No	9 (36.0)	3 (18.7)	
Chronic renal disease			1.00
Yes	3 (12.0)	1 (6.3)	
No	22 (88.0)	15 (93.7)	
Food intake			0.15^d^
Oral	0 (0.0)	2 (12.5)	
Nasogastric tube	24 (96.0)	14 (87.5)	
Gastrostomy	1 (4.0)	0 (0.0)	
Body mass index^b^			0.29
≥ 18.5	20 (80.0)	10 (62.5)	
< 18.5	5 (20.0)	6 (37.5)	

^a^The median age in the control group was used as the cutoff for dividing the residents into two age groups; ^b^The body mass index of 18.5 was used as the cutoff for dividing the residents with and without underweight; ^c^Fisher’s exact test; ^d^Oral vs. non-oral (nasogastric tube + gastrostomy) by Fisher’s exact test

Table 2 shows that, after completing the POCI, the intervention group had significant decreases in PI (1.66 ± 0.78 vs. 0.94 ± 0.64, *p* <  0.01), GI (2.36 ± 0.76 vs. 1.65 ± 0.83, *p* <  0.01), and TCI (0.96 ± 1.10 vs. 0.16 ± 0.47, *p* <  0.01). In contrast, the control group had significant increases in PI and TCI but had no significant change in GI.

**Table 2:** Oral health status of intervention and control groups

**Oral health status**	**Intervention (*N*** **= 25)**	**Control (*N*** **= 16)**	
	**Mean ± SD**	**Mean ± SD**	***p*-value**^b^
Plaque index (PI)			
Before	1.66 ± 0.78	1.54 ± 0.67	0.83
After	0.94 ± 0.64	1.99 ± 0.52	< 0.01
Difference	−0.73 ± 0.43	0.46 ± 0.40	
*p*-value^a^	< 0.01	0.03	
Gingival index (GI)			
Before	2.36 ± 0.76	2.78 ± 0.44	0.14
After	1.65 ± 0.83	2.94 ± 0.17	< 0.01
Difference	−0.70 ± 0.66	0.17 ± 0.35	
*p*-value^a^	< 0.01	0.50	
Tongue coating index (TCI)			
Before	0.96 ± 1.10	0.44 ± 0.96	0.07
After	0.16 ± 0.47	1.13 ± 0.72	< 0.01
Difference	−0.80 ± 0.87	0.69 ± 0.95	
*p*-value^a^	< 0.01	0.02	

^a^Wilcoxon signed-rank test; ^b^Wilcoxon rank-sum test

Table 3 further reveals that, before POCI, total salivary bacterial concentrations were significantly higher in the intervention group compared to the control group ([4.27 ± 3.65] × 10^5^ vs. [2.66 ± 3.32] × 10^5^, *p* = 0.04). After POCI, however, bacterial concentrations in both salivary and sputum samples were significantly lower in the intervention group compared to the control group. In the intervention group, salivary bacterial concentrations significantly (*p* <  0.01) decreased from [4.27 ± 3.65] × 10^5^ before POCI to [0.75 ± 1.20] × 10^5^ after POCI. In the control group, however, salivary bacterial concentration did not significantly differ after POCI. Table 3 shows that both groups had increased bacterial concentrations in sputum samples after the intervention, but the changes were not statistically significant. After undergoing POCI, the intervention group also revealed slower increases in sputum bacterial concentrations compared to the control group.

**Table 3:** Salivary and sputum bacterial concentrations, distribution of *Staphylococcus aureus*, and pneumonia hospitalization status: differences between the intervention group and control group

	Intervention (***N*** = 25)				Control (***N*** = 16)				
	Mean ± SD	Min	Mdn	Max	Mean ± SD	Min	Mdn	Max	***p***-value^c^
**Bacterial concentrations (×10**^**5**^**CFU/mL)**									
Saliva									
Before	4.27 ± 3.65	0.56	3.30	14.26	2.66 ± 3.32	0.01	1.52	12.99	0.04
After	0.75 ± 1.20	< 0.01	0.30	5.09	4.74 ± 9.53	0.08	1.23	33.43	< 0.01
Difference	−3.52 ± 3.37				2.08 ± 10.49				
*p*-value^b^	< 0.01				0.63				
Sputum									
Before	0.59 ± 0.74	0.04	0.34	3.49	1.11 ± 1.63	< 0.01	0.41	5.70	0.64
After	16.59 ± 75.99	< 0.01	0.35	381.06	57.94 ± 226.08	0.16	1.07	905.72	0.04
Difference	16.00 ± 76.08				56.83 ± 226.38				
*p*-value^b^	0.72				0.53				
**Distribution of*Staphylococcus aureus*(%)**^a^									
Saliva									
Before	11.11 ± 14.47	0.00	6.25	41.67	9.03 ± 11.31	0.00	4.17	25.00	0.81
After	1.74 ± 3.75	0.00	0.00	12.50	1.39 ± 2.15	0.00	0.00	4.17	0.86
Difference	−9.38 ± 12.33				−7.64 ± 12.75				
*p*-value^b^	0.02				0.31				
Sputum									
Before	14.17 ± 23.29	0.00	4.17	87.50	22.66 ± 27.89	0.00	12.50	83.33	0.39
After	5.17 ± 10.98	0.00	0.00	50.00	5.21 ± 9.80	0.00	0.00	33.33	0.91
Difference	−9.00 ± 25.16				−17.40 ± 32.96				
*p*-value^b^	0.23				0.07				
**Pneumonia hospitalization**									
Annual prevalence									
Before	1.24 ± 1.51	0.00	1.00	5.00	1.06 ± 1.06	0.00	1.00	3.00	1.00
After	0.48 ± 0.59	0.00	0.00	2.00	1.00 ± 1.21	0.00	1.00	3.00	0.28
Difference	−0.76 ± 1.36				−0.06 ± 0.21				
*p*-value^b^	0.01				1.00				
Hospitalization duration (days)									
Before	10.44 ± 10.95	0.00	10.00	40.00	8.25 ± 8.83	0.00	9.00	32.00	0.52
After	6.14 ± 7.49	0.00	0.00	19.00	5.29 ± 6.41	0.00	7.00	22.00	0.73
Difference	−4.30 ± 11.49				−2.96 ± 2.00				
*p*-value^b^	0.13				0.20				

Mdn: Median; CFU: Colony-forming unit; ^a^(%): percentage of *S. aureus* in 24 randomly and originally selected single colonies; ^b^Wilcoxon signed-rank test; ^c^Wilcoxon rank-sum test

Table 3 shows that, before POCI, the two group did not significantly differ in *S. aureus* distributions in 24 randomly selected single colonies from samples of saliva and sputum. After POCI, however, *S. aureus* distributions in salivary samples significantly decreased in the intervention group (11.11 ± 14.47% vs. 1.74 ± 3.75%, *p* = 0.02) but not in the control group (9.03 ± 11.31% vs. 1.39 ± 2.15%, *p* = 0.31). Meanwhile, *S. aureus* distributions in 24 randomly selected single colonies from sputum samples did not significantly decrease in either the intervention group or the control group. In the intervention group, bacteria with the top 10 high frequency distributions in 24 randomly and originally selected single colonies from both the salivary and sputum samples were listed in Table S1 and S2, respectively.

After the intervention, the annual prevalence of pneumonia hospitalization significantly decreased in the intervention group (1.24 ± 1.51 vs. 0.48 ± 0.59, *p* = 0.01) but not in the control group (Table 3). Neither group showed significant changes in the duration of pneumonia hospitalization. Finally, bacterial concentrations in saliva and sputum samples from the intervention group were classified as high and low according to the median values. As Table 4 shows, a high salivary bacteria concentration after the intervention was significantly associated with pneumonia hospitalization (*p* = 0.04 in Fisher's exact test). Sputum bacteria concentration, however, had no significant association with pneumonia hospitalization.

**Table 4:** Post-intervention correlation between bacterial concentrations and pneumonia hospitalization in the intervention group

Bacterial concentrations	Pneumonia hospitalization		
	Yes	No	
	N (%)	N (%)	***p***-value^b^
Saliva^a^			
High	8 (66.67)	4 (33.33)	0.04
Low	3 (23.08)	10 (76.92)	
Sputum^a^			
High	5 (41.67)	7 (58.33)	1.00
Low	6 (46.15)	7 (53.85)	

^a^The median values for salivary and sputum samples were used to classify the bacterial concentrations as “high” or “low”; ^b^Fisher’s exact test

## Discussion

As reported previously, this study revealed that POCI and improvements in oral health may prevent pneumonia, particularly in NHRs [3, 10, 16]. Improved oral hygiene and decreased concentrations of oral pathogenic microorganisms can decrease dental plaque accumulation and bacterial pneumonia risk [10, 16, 31]. Pneumonia rates are known to be significantly lower in NHRs with good oral health compared to those with poor oral health [10, 16]. One intervention study also reported that the pneumonia occurrence rate significantly differed between NHRs with and without POCI (0.45% vs. 1.20%, *p* = 0.006) [3]. In accordance with the literature, NHRs who had completed the POCI in the present study had a significantly lower annual prevalence of pneumonia hospitalization compared to the control group without POCI. Additionally, salivary bacterial concentration had a significant positive correlation with pneumonia hospitalization.

Nursing home residents often have multiple diseases, systemic diseases, and malnourishment, which can prolong their hospital stay [32]. A previous study reported that intensive care units (ICUs) tend to neglect oral hygiene in patients with unstable vital signs [33]. A Brazil study also reported that good oral hygiene in ICU patients is associated with a shorter ICU stay and a lower risk of complications [32]. In the present study, POCI was not significantly associated with a shorter duration of pneumonia hospitalization. However, the POCI was not implemented at the time of hospital admission for pneumonia, which may partially explain why the duration of pneumonia hospitalization did not significantly decrease.

Many studies agree that oral care for NHRs can significantly improve salivary bacterial concentration, PI, GI, and TCI [34, 35]. Our results agree with these findings. Sputum contains secretions from the lower respiratory tract, nose, mouth, and pharynx as well as cellular debris and microorganisms [36]. Although both groups in the present study showed increased sputum bacterial concentrations, the increase was larger in the control group. Dental hygienists who treated NHRs in the intervention group were required to use a suction tool to remove contaminated liquid from the mouth. A previous study reported that a significant portion of sputum (up to 70%) could be contaminated by bacteria in the saliva sample [37]. In the intervention group, the major contaminants were removed with a suction tool after POCI. Therefore, we suspect that the release of minor residual contaminants from the oral cavity into the larynx caused a slight increase in the sputum bacterial concentration. In contrast, since the suction tool was not used in the control group, the amount of bacteria released from the oral cavity into the sputum was higher in comparison with the intervention group. That is, the use of a suction tool in the intervention group may explain why the two groups had different increases in sputum bacterial concentrations.

The TCI is an essential indicator of oral hygiene because a high TCI indicates an elevated salivary bacterial concentration, which can cause pneumonia [35]. In the present study, a high TCI was not significantly associated with a high salivary bacterial concentration. A possible explanation is that all residents had low TCI scores (Mean ± SD = 0.76 ± 1.07). Dental plaque accumulation was another important cause of increased salivary bacterial concentration [10]. In the present study, salivary bacterial concentration was significantly higher in participants with high TCI and high PI compared to those with low TCI and low PI. Therefore, we believed that increased TCI may have partially contributed to the elevated salivary bacterial concentration and the development of pneumonia in the NHRs in this study. Notably, the nursing home caregivers did not care tongue coating index during regular oral cleaning. After undergoing POCI, NHRs in this study had a lower TCI. A Japan study previously reported that tongue cleaning can reduce oral pathogens and prevent pneumonia [35]. Taken together, these results suggest that tongue cleaning should be included in routine oral care protocols for NHRs.

Salivary gland massage can induce secretion of saliva [3, 38]. Positive effects of increased secretion of saliva include reduction of oral pathogens and oral bacteria attached to tooth surfaces [38]. Previous studies reported that *S. aureus* infection in the oral cavity as an important risk factor for pneumonia [39, 40]. In the present study, POCI significantly decreased the total salivary bacterial concentration as well as the distribution of *S. aureus* in salivary samples. Although salivary secretion rate was not measured in this study, dental hygienists did observe that salivary gland massage increased the moistness of oral mucosa but decreased plaque accumulation in the oral cavity. Therefore, the decline in the annual prevalence of pneumonia hospitalization was likely attributable to reductions in total bacteria and in the distribution of *S. aureus* in salivary samples after POCI.

Previous studies in Japan have found that an oral care intervention provided once a week by dentists and dental hygienists for NHRs can effectively decrease both oral bacterial concentration and pneumonia mortality rate [16, 41]. Another study in the United States reported that an oral care intervention delivered twice a day by well-trained nursing home staff could reduce the pneumonia mortality rate in NHRs [42]. The present study found that POCI once a week can substantially reduce the annual prevalence of pneumonia hospitalization but not the duration of pneumonia hospitalization. Therefore, low interventional frequency and poor oral care quality might tend to increase the duration of pneumonia hospitalization. Further research is warranted to analyze the effects of intensive oral care, which may reveal a significant correlation between the duration of pneumonia hospitalization and oral care.

The present study had some limitations. First, the NHRs in this study were only recruited from two local nursing homes. The sample size was small; thus, the results cannot be reliably extrapolated to other regions or populations. Second, epidemiological data indicate that aspiration pneumonia accounts for 5 to 15% of pneumonia cases in hospitalized populations [43] and that bacterial infection is the underlying cause of pneumonia in approximately 40% of NHRs with pneumonia [44]. In this study, nursing home staff members did not record episodes of aspiration pneumonia; thus, the exact role of POCI in preventing aspiration pneumonia could not be determined. A further large-scale well-designed study would help to distinguish the effects of POCI on aspiration and nonaspiration pneumonia in NHRs. Third, *S. aureus* concentrations were not determined in the participants. The effect of *S. aureus* concentration on pneumonia hospitalization might be underestimated. The distribution of *S. aureus* in 24 randomly selected single colonies in each salivary sample could partially reflect the proportion of *S. aureus* to total salivary bacteria count. The *S. aureus* distribution in each salivary sample is a possible surrogate for predicting the *S. aureus* concentration. Finally, pneumonia can be caused by different critical pathogenic species. The bacterial culture method in this study only cultured a limited number of bacterial strains. Some potentially fatal pneumonia pathogens were not investigated. Therefore, further studies are needed to identify other critical pathogenic species of pneumonia.

In conclusion, our results indicate that, in NHRs, POCI can significantly improve oral health, reduce salivary bacterial concentration, decrease the distribution of *S. aureus*, and decrease the pneumonia hospitalization prevalence rate, but it does not affect the duration of hospitalization for pneumonia or sputum bacterial concentration.

## Supplementary information

**Additional file 1 Table S1** Frequency distributions of top 10 bacterial species identified in 24 randomly and originally selected single colonies in salivary samples from intervention group **Table S2** Frequency distributions of top 10 bacterial species identified in 24 randomly and originally selected single colonies in sputum samples from intervention group

## Data Availability

The datasets are available from the corresponding author upon reasonable request.

## Abbreviations

CFU: Colony-forming units
GI: Gingival index
KMUH: Kaohsiung Medical University Hospital
MALDI-TOF MS: Matrix-assisted laser desorption/ionization–time of flight mass spectrometer
Mdn: Median
NHRs: Nursing home residents
PI: Plaque index
POCI: Professional oral care intervention
RT-PCR: Real-time polymerase chain reaction
*S. aureus*: *Staphylococcus aureus*
TCI: Tongue coating index
