# Supplementary material for: The effect of oral care intervention on pneumonia hospitalization, Staphylococcus aureus distribution, and salivary bacterial concentration in Taiwan nursing home residents: a pilot study
Source: BMC Infect Dis. 2020 May 27;20:374. doi: 10.1186/s12879-020-05061-z (PMC7251833; doi:10.1186/s12879-020-05061-z)
Supplement: Supplementary file 1 — Additional file 1 Table S1 Frequency distributions of top 10 bacterial species identified in 24 randomly and originally selected single colonies in salivary samples from intervention group Table S2 Frequency distributions of top 10 bacterial species identified in 24 randomly and originally selected single colonies in sputum samples from intervention group [file 12879_2020_5061_MOESM1_ESM.docx]

**Table S1** Frequency distributions of top 10 bacterial species identified in 24 randomly and originally selected single colonies in salivary samples from intervention group

| **Bacterial species** | **%** | **Type†** |
| --- | --- | --- |
| **Pre-test** |  |  |
| Gram-positive |  |  |
| *Corynebacterium striatum* | 16.0 | Aerobe |
| *Rothia mucilaginosa* | 11.1 | Aerobe |
| *Staphylococcus aureus** | 11.1 | Aerobe |
| *Streptococcus mitis* | 7.1 | Facultative anaerobe |
| *Streptococcus agalactiae* | 6.7 | Facultative anaerobe |
| *Streptococcus oralis* | 4.7 | Facultative anaerobe |
| *Streptococcus dysgalactiae* | 4.1 | Facultative anaerobe |
| *Streptococcus pneumoniae** | 1.4 | Facultative anaerobe |
| *Streptococcus constellatus* | 1.2 | Anaerobe |
| *Corynebacterium amycolatum* | 0.6 | Aerobe |
| Gram-negative |  |  |
| *Pseudomonas aeruginosa** | 8.7 | Facultative anaerobe |
| *Acinetobacter baumannii** | 4.7 | Aerobe |
| *Proteus mirabilis* | 4.3 | Facultative anaerobe |
| *Neisseria mucosa* | 3.4 | Aerobe |
| *Neisseria flavescens* | 2.0 | Aerobe |
| *Providencia stuartii* | 1.4 | Facultative anaerobe |
| *Aggregatibacter segnis* | 1.2 | Facultative anaerobe |
| *Neisseria perflava* | 1.2 | Aerobe |
| *Haemophilus haemolyticus* | 1.0 | Facultative anaerobe |
| *Pasteurella multocida* | 1.0 | Facultative anaerobe |
| Others | 7.1 | - |
| **Post-test** |  |  |
| Gram-positive |  |  |
| *Streptococcus mitis* | 15.5 | Facultative anaerobe |
| *Rothia mucilaginosa* | 13.1 | Aerobe |
| *Corynebacterium striatum* | 8.3 | Aerobe |
| *Streptococcus dysgalactiae* | 7.2 | Facultative anaerobe |
| *Streptococcus agalactiae* | 4.3 | Facultative anaerobe |
| *Streptococcus oralis* | 4.3 | Facultative anaerobe |
| *Streptococcus pneumoniae** | 1.7 | Facultative anaerobe |
| *Staphylococcus aureus** | 1.7 | Aerobe |
| *Streptococcus constellatus* | 1.1 | Anaerobe |
| *Streptococcus anginosus* | 0.7 | Anaerobe |
| Gram-negative |  |  |
| *Pseudomonas aeruginosa** | 5.2 | Facultative anaerobe |
| *Providencia stuartii* | 5.0 | Facultative anaerobe |
| *Neisseria flavescens* | 4.3 | Aerobe |
| *Kerstersia gyiorum* | 3.4 | Aerobe |
| *Acinetobacter baumannii** | 2.5 | Aerobe |
| *Neisseria mucosa* | 2.5 | Aerobe |
| *Aggregatibacter segnis* | 2.3 | Facultative anaerobe |
| *Neisseria cinerea* | 1.7 | Aerobe |
| *Proteus mirabilis* | 1.7 | Facultative anaerobe |
| *Aggregatibacter aphrophilus* | 0.9 | Facultative anaerobe |
| Others | 12.6 | - |

*Bacteria recognized as pneumonia pathogen; †Data verified at “https://www.atcc.org”.

**Table S2** Frequency distributions of top 10 bacterial species identified in 24 randomly and originally selected single colonies in sputum samples from intervention group

| **Bacterial species** | **%** | **Type**† |
| --- | --- | --- |
| **Pre-test** |  |  |
| Gram-positive |  |  |
| *Staphylococcus aureus** | 14.2 | Aerobe |
| *Rothia mucilaginosa* | 12.4 | Aerobe |
| *Corynebacterium striatum* | 6.3 | Aerobe |
| *Streptococcus agalactiae* | 2.6 | Facultative anaerobe |
| *Streptococcus dysgalactiae* | 2.6 | Facultative anaerobe |
| *Streptococcus mitis* | 2.2 | Facultative anaerobe |
| *Streptococcus oralis* | 2.2 | Facultative anaerobe |
| *Leuconostoc lactis* | 0.6 | Facultative anaerobe |
| *Streptococcus constellatus* | 0.6 | Anaerobe |
| *Actinomyces odontolyticus* | 0.4 | Facultative anaerobe |
| Gram-negative |  |  |
| *Proteus mirabilis* | 12.9 | Facultative anaerobe |
| *Providencia stuartii* | 10.3 | Facultative anaerobe |
| *Pseudomonas aeruginosa** | 9.0 | Facultative anaerobe |
| *Morganella morganii* | 6.5 | Facultative anaerobe |
| *Neisseria mucosa* | 5.4 | Aerobe |
| *Acinetobacter baumannii** | 3.5 | Aerobe |
| *Haemophilus influenzae** | 1.3 | Facultative anaerobe |
| *Moraxella catarrhalis* | 0.9 | Anaerobe |
| *Serratia marcescens* | 0.7 | Facultative anaerobe |
| *Serratia ureilytica* | 0.7 | Facultative anaerobe |
| Others | 4.7 | - |
| **Post-test** |  |  |
| Gram-positive |  |  |
| *Rothia mucilaginosa* | 16.8 | Aerobe |
| *Streptococcus oralis* | 7.8 | Facultative anaerobe |
| *Streptococcus mitis* | 5.8 | Facultative anaerobe |
| *Staphylococcus aureus** | 5.2 | Aerobe |
| *Corynebacterium striatum* | 5.2 | Aerobe |
| *Streptococcus dysgalactiae* | 2.0 | Facultative anaerobe |
| *Streptococcus salivarius* | 1.5 | Facultative anaerobe |
| *Streptococcus agalactiae* | 1.3 | Facultative anaerobe |
| *Agromyces mediolanus* | 0.8 | Aerobe |
| *Streptococcus anginosus* | 0.5 | Anaerobe |
| Gram-negative |  |  |
| *Proteus mirabilis* | 15.0 | Facultative anaerobe |
| *Providencia stuartii* | 13.8 | Facultative anaerobe |
| *Neisseria mucosa* | 5.2 | Aerobe |
| *Pseudomonas aeruginosa** | 2.5 | Facultative anaerobe |
| *Acinetobacter baumannii** | 2.3 | Aerobe |
| *Morganella morganii* | 2.3 | Facultative anaerobe |
| *Pasteurella multocida* | 1.8 | Facultative anaerobe |
| *Serratia marcescens* | 1.3 | Facultative anaerobe |
| *Providencia rettgeri* | 1.0 | Facultative anaerobe |
| *Aggregatibacter segnis* | 0.8 | Facultative anaerobe |
| Others | 7.1 | - |

*Bacteria recognized as pneumonia pathogen; †Data verified at “https://www.atcc.org”.
